# Traffic-Generated Changes in the Chemical Characteristics of Size-Segregated Urban Aerosols

**DOI:** 10.1007/s00128-014-1364-9

**Published:** 2014-08-29

**Authors:** Wioletta Rogula-Kozłowska

**Affiliations:** Institute of Environmental Engineering, Polish Academy of Sciences, 34 M. Skłodowska-Curie St., 41-819 Zabrze, Poland

**Keywords:** Vehicular traffic, Particulate matter, Chemical composition, Ultrafine particles, PAHs

## Abstract

The road traffic impact on the concentrations of 13 fractions of particulate matter (PM) and their components was assessed. PM was sampled at two points in Katowice (southern Poland), a background point beyond the effects of road traffic, and a near-highway traffic point. The samples were analyzed for organic and elemental carbon, 8 water-soluble ions, 24 elements, and 16 polycyclic aromatic hydrocarbons (PAHs). The traffic emissions (mainly particles from car exhaust) enriched the ultrafine, submicron, and fine PM particles with elemental carbon. The traffic-caused re-suspension of the road and soil dust affecting the concentrations and chemical composition of the coarse PM fraction. However, for each PM fraction, the carcinogenic equivalent ratios, assumed as a measure of the hazard from 16 PAHs in this paper, were similar at the two sampling points. The traffic emissions from the highway appeared to have a weaker influence on the concentrations and chemical composition of PM in a typical urban area of southern Poland than elsewhere in Europe.

The growth of road traffic causes the growth of health hazards from atmospheric aerosols (Schwartz 1997; Han and Naeher 2006). However, attributing this effect only to the traffic-related elevation of particulate matter (PM) concentrations, although most obvious, is a simplification–in great part the threat is due to the specific influence of traffic emissions on the chemistry of PM (Han and Naeher 2006; Daher et al. 2014). In general, the ambient concentrations of PM-bound carbonaceous matter (including polycyclic aromatic hydrocarbons, elemental and organic carbon) are higher at crossroads and highways than in areas beyond the traffic influence (e.g. Harrison et al. 2004; Hueglin et al. 2005; Slezakova et al. 2010). The concentrations of PM-bound sulfates, nitrates, ammonium, chlorates, soil matter, and some elements at traffic-affected sites apparently differ from those in rural, urban, or suburban areas where the traffic effects are weaker (Harrison et al. 2004; Hueglin et al. 2005; Daher et al. 2014).

The goal of the work was to determine the differences in the chemical composition between various fractions of PM and to evaluate the road traffic influence on these differences in a typical urban area in southern Poland. PM was sampled in Katowice, at two sites differing in the traffic contributions to the air pollution. The concentrations, chemical composition, and exactness of the mass reconstruction (chemical mass closure) of each of thirteen following PM fractions: PM_0.03-0.06_, PM_0.06-0.108_, PM_0.108-0.17_, PM_0.17-0.26_, PM_0.26-0.4_, PM_0.4-0.65_, PM_0.65-1_, PM_1-1.6_, PM_1.6-2.5_, PM_2.5-4.4_, PM_4.4-6.8_, PM_6.8-10_, PM_10-40_ (subscript ranges are the intervals of the particle aerodynamic diameters D_p_ in µm) at these two sites are discussed in the paper.

## Materials and Methods

**Fig. 1:**
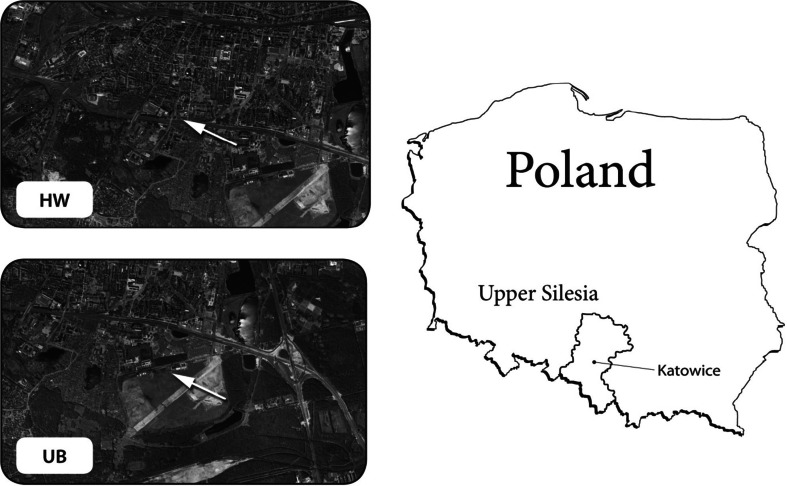
Location of the measuring sites in Katowice

Particulate matter was sampled simultaneously at two points in Katowice, a city in southern Poland with approximately 2,000,000 population. One of the points, UB (Fig. 1), was an urban background sampling point (2008/50/EC Directive), and was located beyond the effects of industrial and traffic emissions (the nearest road was 150 m away, and traffic density was less than 2,500 cars per 24 h). The area was a living district surrounded by greenbelts, about 2,000 m from the city center. The meteorological conditions at the point were typical of the region. The second point, HW (Fig. 1), was a traffic point located on the shoulder of the highway A4, about 1,500 m south of the city center and 1,200 m northwest of the point UB. Average traffic density at HW was 30,000 cars per 24 h. To the north of HW, there was a living quarter. To the south, there was an open area of the A4 highway, and further behind A4, about 300 m from the point, there were some public utilities and detached houses of another living quarter. PM was sampled with the use of two thirteen-stage low pressure DLPI impactors (Dekati Ltd, Kangasala, FL).

In Katowice, especially in winter, traffic PM emissions can be obscured by emissions of PM from combustion of fossil fuels or biomass for residential heating (Rogula-Kozłowska et al. 2013; Rogula-Kozłowska and Klejnowski 2013). Therefore, the sampling period was selected beyond the major heating season, and the samples were taken within mid-March to mid-June of 2012. There were nine, about one-week long (from 142 to 173 h), pairwise sample-takings.

The mass of the collected PM was determined by weighing the substrates before and after the exposure; a MYA 5.3Y.F micro balance (RADWAG; Radom, PL) was used (1 µg resolution). Before each weighing, the substrates were conditioned for 48 h in the weighing room (relative air humidity 45 % ± 5 %, air temperature 20 ± 2°C). The substrates and impactors were prepared for exposure in a laminar chamber. After weighing, the exposed substrates were stored in a freezer until analysis.

Two kinds of Whatman (GE Healthcare Bio-Sciences Corp.; Piscataway, NJ, USA) substrate filters were used. Alternating between the sample-takings, quartz fiber filters (QMA, ø25 mm, CAT No. 1851-025) and nylon membrane filters (0.2 µm, ø25 mm, Cat No. 7402-002) were used; the former four and the latter five times. Always the same type substrates were used on all impactor stages, with the same type simultaneously used at both sampling points. Altogether, nine samples (four on quartz and five on nylon filters) of each of the 13 PM fractions were taken at each point over the entire measuring period.

At each point, four samples of each fraction were taken on quartz filters. Two equal (1.5 cm^2^) fragments were cut out from each quartz filter just before the analysis. The PM on one of them was analyzed for organic carbon (OC) and elemental carbon (EC). The remaining fragments, four for each of the 13 fractions and each point, were used to make fraction samples. A fraction sample for a point was made by putting together the four filter fragments containing this PM fraction sample from this point. Each of these 26 fraction samples was analyzed for 16 PAHs.^1^

Five samples of each fraction were taken on nylon filters at each sampling point. The PM on each nylon filter was analyzed for elemental composition first (i.e. for Al, Si, Sc, Ti, V, Cr, Mn, Fe, Co, Ni, Cu, Zn, As, Se, Br, Rb, Sr, Mo, Ag, Cd, Sb, Te, Ba, Pb), then it was extracted in water and concentrations of the main ions (Cl^−^, NO_3_^−^, SO_4_^2−^, Na^+^, NH_4_^+^, K^+^, Ca^2+^, Mg^2+^) were determined in the extracts.

The OC and EC contents of dust were determined with the use of a Lab OC–EC Aerosol Analyzer (Sunset Laboratories Inc.; Portland, OR, USA). Sixteen PAHs^1^ in the PM fractions were determined on a Clarus 500 gas chromatograph (PerkinElmer; Waltham, MA, USA). The analytical procedures, equipment, and quality control for OC, EC, and PAH in PM are described in Rogula-Kozłowska and Klejnowski (2013) and Rogula-Kozłowska et al. (2013).

The elemental composition of PM was determined by means of energy dispersive X-ray fluorescence (EDXRF). An Epsilon 5 instrument (PANalytical B.V.; Almelo, NL), calibrated with thin-layer single-element standards (Micromatter; Vancouver, BC,CA) was used to measure total concentrations of the elements. To control the performance of the analytical procedure, samples of reference material (SRM2873, NIST, Gaithersburg, MD, USA) were measured weekly. The recoveries were between 85 % and 120 % of the certified values (except 52 % and 39 % recoveries of V and Co) and the X-ray tube and detector drifts were monitored monthly. The detection limits were from 0.18 ng/cm^2^ (Se) to 19.6 ng/cm^2^ (Si).

The water extracts of PM were made by ultrasonizing the substrates containing the samples in 25 cm^3^ of de-ionized water for 60 min at 15°C, then shaking for about 12 h (18°C, 60 r/min). The ion content of extracts was determined using an ion chromatograph (Metrohm AG; Herisau, CH). The method was validated against the CRM Fluka products nos. 89316 and 89886; the standard recoveries were 92 %–109 % of the certified values, and the detection limits were: 10 ng/cm^3^ for NH_4_^+^, 18 ng/cm^3^ for Cl^−^, SO_4_^2−^ and K^+^, 27 ng/cm^3^ for NO_3_^−^ and Na^+^, and 36 ng/cm^3^ for Ca^2+^ and Mg^2+^.

## Results and Discussion

**Fig. 2:**
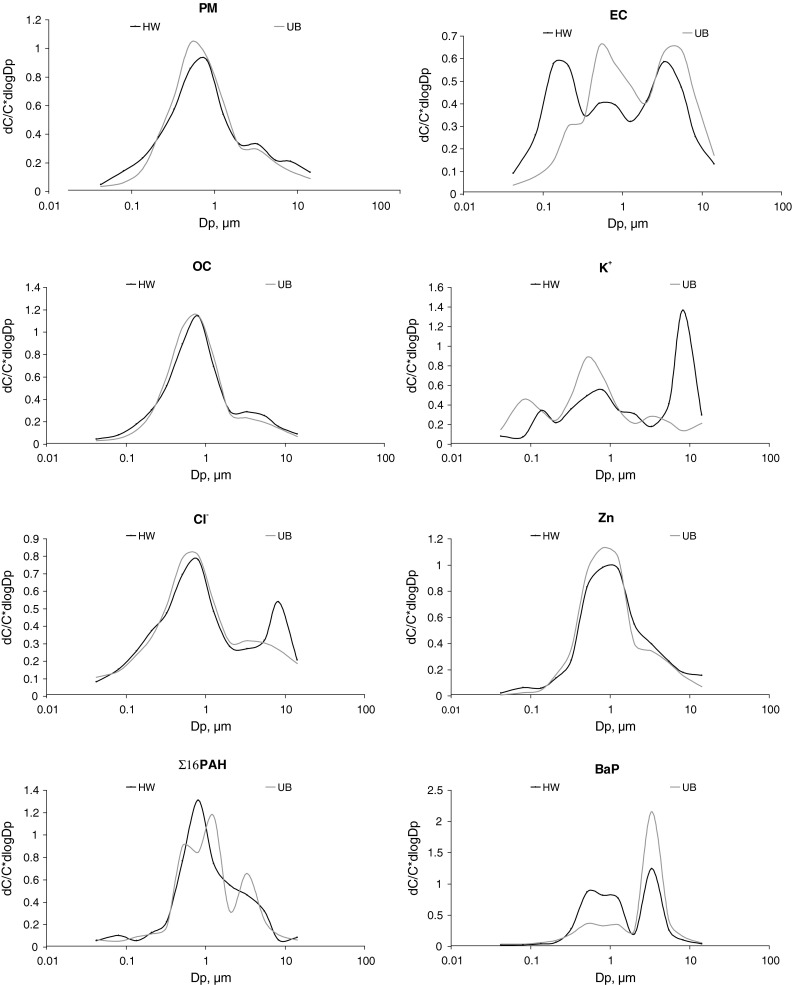
Mass size distribution of PM and selected components of PM at urban background (UB) and at the highway (HW)

The average concentrations for the sampling period are presented in Table 1. In Katowice, the core PM mass consisted of particles with D_p_ between 0.17 and 1.6 µm (PM_0.17-1.6_). They contributed 72 % to the total PM mass at UB and 64 % at HW (Table 1). The density function of the PM mass size distribution assumes the absolute maximum between 0.4 and 1.0 µm at both sampling points (Fig. 2). It is clear that the concentrations of PM_0.17-1.6_ were not significantly affected by road traffic.

**Table 1:** Average concentrations of PM, PM-bound OC, EC (µg/m^3^), water soluble ions, and the remaining PM components (ng/m^3^) of 13 PM fractions at the highway (HW) and urban background (UB) sampling stations

	>10	6.8–10	4.4–6.8	2.5–4.4	1.6–2.5	1–1.6	0.65–1	0.4–0.65	0.26–0.4	0.17–0.26	0.108–0.17	0.06–0.108	0.03–0.06
*HW*													
PM	1.205	1.056	1.225	2.434	1.917	3.286	5.122	5.410	3.215	2.088	1.383	1.090	0.436
OC	0.246	0.234	0.427	0.628	0.513	1.255	1.898	1.675	0.868	0.500	0.316	0.188	0.119
EC	0.067	0.071	0.149	0.239	0.133	0.109	0.123	0.141	0.109	0.174	0.189	0.111	0.046
Na^+^	16.168	28.878	17.830	24.918	17.768	11.708	10.875	10.031	7.851	3.450	4.190	1.256	0.673
NH_4_^+^	7.513	10.260	9.098	19.862	46.384	188.273	319.510	321.417	154.761	82.144	32.228	19.260	9.304
K^+^	11.778	30.140	10.249	5.837	7.973	9.423	13.618	13.707	8.867	5.367	8.946	2.500	3.308
Ca^2+^	10.923	18.521	13.642	13.111	1.939	0.819	1.126	8.284	5.711	2.413	2.509	3.233	0.885
Mg^2+^	0.000	0.000	0.000	0.000	0.000	0.000	0.000	1.013	0.724	0.000	0.000	0.000	0.836
Cl^−^	50.804	73.618	49.083	54.308	45.010	80.622	118.662	119.241	72.414	56.103	41.571	31.888	20.092
NO_3_^−^	32.948	37.912	56.257	112.289	122.387	239.734	375.192	339.463	173.948	112.485	56.015	34.574	21.951
SO_4_^2−^	46.012	42.225	48.790	75.657	97.431	262.982	460.111	489.314	237.160	117.883	62.055	43.623	27.034
Al	11.046	7.115	15.018	21.615	12.148	5.053	1.978	1.947	1.731	1.828	1.750	1.632	1.540
Si	46.386	25.885	55.733	85.388	46.983	25.837	9.361	5.102	1.890	1.631	1.526	1.720	1.753
Sc	4.768	2.858	5.046	7.828	4.012	2.743	0.979	0.464	0.430	0.409	0.469	0.482	0.438
Ti	14.973	14.716	16.144	17.798	17.104	16.398	12.870	12.758	14.584	12.382	13.381	13.968	12.722
V	1.992	1.967	2.194	2.744	2.506	2.434	1.811	1.814	1.919	1.598	1.756	1.772	1.586
Cr	0.745	0.563	0.728	1.097	0.984	0.887	0.798	0.720	0.545	0.619	0.575	0.611	0.608
Mn	5.190	4.884	6.473	7.606	6.521	6.195	4.941	4.550	4.371	3.513	3.822	3.862	3.499
Fe	42.740	28.139	69.467	166.915	122.029	101.696	45.257	25.158	7.863	5.023	2.870	3.348	1.556
Co	0.353	0.372	0.409	0.554	0.551	0.553	0.425	0.265	0.367	0.240	0.283	0.270	0.291
Ni	0.067	0.058	0.109	0.193	0.138	0.150	0.132	0.145	0.055	0.063	0.032	0.038	0.032
Cu	0.836	0.783	1.640	4.300	3.563	3.764	2.418	1.784	0.821	0.602	0.513	0.568	0.377
Zn	3.837	2.447	4.110	7.966	8.726	16.233	15.025	14.328	4.155	2.086	0.955	1.326	0.547
As	0.416	0.307	0.475	0.819	1.417	2.558	2.079	2.184	0.796	0.451	0.620	0.325	0.164
Se	0.007	0.000	0.000	0.003	0.017	0.110	0.129	0.251	0.014	0.000	0.000	0.000	0.000
Br	0.155	0.133	0.157	0.221	0.240	1.168	1.955	2.340	0.860	0.578	0.268	0.290	0.164
Rb	0.018	0.019	0.033	0.061	0.042	0.160	0.257	0.294	0.072	0.076	0.019	0.031	0.016
Sr	0.421	0.329	0.506	0.714	0.502	0.527	0.322	0.325	0.137	0.241	0.264	0.202	0.261
Mo	0.206	0.154	0.257	0.355	0.272	0.289	0.175	0.166	0.139	0.155	0.152	0.153	0.096
Ag	0.277	0.250	0.239	0.275	0.234	0.249	0.324	0.269	0.306	0.272	0.239	0.336	0.230
Cd	0.354	0.414	0.306	0.443	0.471	0.691	0.558	0.579	0.378	0.412	0.403	0.331	0.316
Sb	6.975	7.245	7.438	7.573	8.299	8.746	7.559	7.670	8.304	6.664	7.016	7.259	6.757
Te	0.145	0.161	0.264	0.096	0.188	0.256	0.198	0.196	0.281	0.139	0.344	0.195	0.285
Ba	2.114	1.983	2.679	5.169	4.342	3.697	2.460	1.676	1.700	1.625	1.716	1.707	1.662
Pb	1.192	0.885	1.342	2.371	4.138	7.541	5.857	5.936	2.122	1.251	2.023	0.836	0.429
∑16PAH	0.380	0.154	0.883	1.667	1.568	2.223	3.575	2.363	0.615	0.350	0.167	0.384	0.253
BaP	0.059	0.021	0.152	0.731	0.625	0.573	0.562	0.173	0.071	0.039	0.016	0.036	0.032
^1^CEQ	0.069	0.026	0.201	0.839	0.732	0.610	0.966	0.544	0.087	0.049	0.021	0.044	0.038
*UB*													
PM	0.763	0.666	1.074	2.038	1.734	3.613	4.932	6.147	3.499	2.038	0.867	0.454	0.287
OC	0.176	0.205	0.318	0.494	0.458	1.365	1.845	1.884	0.969	0.448	0.212	0.113	0.081
EC	0.039	0.054	0.091	0.116	0.059	0.074	0.081	0.104	0.046	0.042	0.022	0.015	0.009
Na^+^	10.407	3.075	9.161	26.696	16.255	12.834	9.183	19.527	8.298	4.448	4.632	3.286	2.777
NH_4_^+^	13.831	9.044	20.624	25.501	54.505	179.747	320.046	366.913	164.991	74.832	26.876	13.682	5.191
K^+^	7.757	2.781	5.184	8.516	5.053	9.027	15.749	22.716	11.215	5.490	8.251	14.182	5.548
Ca^2+^	8.548	6.600	3.000	10.952	5.558	0.000	0.000	7.436	11.860	9.259	5.183	4.572	7.212
Mg^2+^	0.000	0.000	0.000	0.000	0.000	0.000	0.000	0.000	0.000	0.000	0.000	0.000	0.000
Cl^−^	43.156	34.199	44.306	59.946	46.084	84.576	115.725	127.896	74.614	47.826	35.863	27.892	25.184
NO_3_^−^	36.007	36.969	60.575	121.791	133.701	246.005	392.430	379.114	181.640	96.612	42.187	28.192	23.170
SO_4_^2−^	44.180	44.027	50.281	81.859	104.316	265.985	450.767	545.011	269.407	146.037	57.457	40.197	28.578
Al	8.226	6.782	14.627	19.193	9.971	4.762	1.527	1.811	1.706	1.565	1.857	1.759	2.081
Si	31.860	22.769	51.817	72.083	34.905	24.397	8.217	5.330	2.041	1.508	1.477	1.745	1.991
Sc	2.258	2.596	4.844	6.719	2.810	2.160	0.682	0.351	0.399	0.404	0.449	0.502	0.451
Ti	14.313	15.253	15.993	17.598	16.872	14.282	14.127	12.871	13.584	12.787	13.806	13.343	13.925
V	1.907	2.058	2.164	2.428	2.346	2.010	1.929	1.804	1.760	1.664	1.786	1.694	1.747
Cr	0.683	0.639	0.669	0.919	0.705	0.744	0.770	0.665	0.580	0.557	0.656	0.514	0.558
Mn	4.357	4.814	5.971	7.369	5.878	5.653	5.331	4.671	3.963	3.664	3.735	3.738	3.864
Fe	27.140	21.897	57.477	95.929	51.490	62.558	25.442	17.319	7.332	4.293	1.985	1.795	1.320
Co	0.276	0.326	0.421	0.401	0.408	0.357	0.340	0.252	0.312	0.341	0.263	0.318	0.282
Ni	0.061	0.051	0.090	0.131	0.077	0.127	0.104	0.124	0.076	0.052	0.038	0.035	0.026
Cu	0.582	0.685	1.519	2.201	1.981	3.511	3.308	2.820	1.162	0.764	0.436	0.420	0.337
Zn	1.572	1.902	3.506	6.200	5.738	15.902	15.607	14.747	4.973	2.249	0.639	0.459	0.182
As	0.408	0.356	0.496	1.020	1.078	2.904	2.361	2.205	1.000	0.604	0.295	0.375	0.291
Se	0.000	0.000	0.000	0.007	0.000	0.125	0.152	0.233	0.039	0.000	0.000	0.000	0.000
Br	0.117	0.165	0.207	0.234	0.313	1.066	2.054	2.707	1.274	0.765	0.403	0.648	0.575
Rb	0.022	0.019	0.039	0.064	0.033	0.139	0.283	0.387	0.153	0.072	0.028	0.049	0.049
Sr	0.282	0.282	0.517	0.609	0.390	0.367	0.290	0.264	0.288	0.248	0.291	0.277	0.331
Mo	0.092	0.098	0.155	0.201	0.185	0.170	0.185	0.283	0.182	0.129	0.143	0.188	0.075
Ag	0.243	0.317	0.272	0.294	0.318	0.290	0.329	0.300	0.227	0.273	0.325	0.292	0.333
Cd	0.388	0.383	0.431	0.369	0.356	0.741	0.531	0.622	0.555	0.370	0.487	0.399	0.365
Sb	6.693	7.593	7.559	7.637	8.190	7.371	8.340	8.054	7.439	7.171	7.139	7.088	7.186
Te	0.285	0.269	0.219	0.279	0.195	0.272	0.175	0.167	0.123	0.120	0.241	0.171	0.143
Ba	1.910	1.920	2.575	3.108	2.386	2.737	1.878	1.654	1.585	1.710	1.559	1.585	1.742
Pb	1.122	0.966	1.439	3.077	3.223	8.648	6.633	5.856	2.733	1.626	0.745	1.063	0.849
∑16PAH	0.289	0.281	0.696	2.461	0.945	3.641	2.411	2.892	0.514	0.333	0.273	0.203	0.300
BaP	0.041	0.062	0.180	1.219	0.113	0.165	0.142	0.178	0.087	0.041	0.028	0.022	0.023
^1^CEQ	0.050	0.072	0.214	1.304	0.160	0.674	0.652	0.782	0.226	0.051	0.034	0.026	0.027

^1^CEQ = 0.001 × [Na + Acy + Ace + F + Ph + Fl + Py] + 0.01 × [An + Ch + BghiP] + 0.1 × [BaA + BbF + BkF + IP] + 1 × [BaP] + 5 × [DBA]

The concentrations of PM_0.17-1.6_ and of its chemical components did not differ between the points except for (usually) traffic-related Fe, Co, Ni, and Mo (Pant and Harrison 2013), and for EC.

In general, the fractionated EC concentrations were higher at HW than at UB. Within PM_0.17-1.6_, the concentrations of PM_0.17-0.26_-, PM_0.26-0.4_-, PM_0.4-0.65_-, PM_0.65-1_-, PM_1-1.6_-bound EC were from 40 % to >300 % higher at HW than at UB. The concentrations of PM_0.03-0.06_, PM_0.06-0.108_, PM_0.108-0.17_-related EC were from 4 to 8 times greater at HW than at UB. The striking difference in the behavior of the concentrations of PM-related EC between HW and UB can be seen by comparing the density functions of the mass distribution (relative to particle size) of EC bound to the nuclei and accumulation modes at HW and UB (Fig. 2).

Also, the concentrations of PM_0.03-0.17_ and of PM_0.03-0.17_-bound OC were higher at HW than at UB (averages of the concentrations of PM_0.03-0.06_, PM_0.06-0.108_, PM_0.108-0.17_, and of the related OC were higher by 84 % and 54 %, respectively). The concentrations of PM_0.03-0.17_-related Fe, Cu, and Zn were also elevated at HW. The density functions for some PM-related metals (e.g. Zn, Fig. 2) had maxima in the interval 0.03-0.17 µm at HW; these maxima did not exist at UB. Great differences in the concentrations of PM_0.03-0.06_-, PM_0.06-0.108_-, and PM_0.108-0.17_-bound EC, OC, Fe, Cu, and Zn between the points are due to the chemical composition of car exhaust: car exhaust consists mainly of ultrafine particles of EC, OC (incomplete fuel combustion), substances synthesized from residues of fuel combustion, and lubricant or fuel additives (metals and their compounds, e.g.) (Geller et al. 2006; Maricq 2007).

Surprisingly, despite the significant PAH content of exhaust OC, the ambient concentrations of fine particle-bound ∑PAH did not differ significantly between the points except for the PM_0.06-0.108_-bound ∑PAH, higher at HW than at UB by 90 %. The density function of the PM-bound ∑PAH distribution had the maximum in 0.06-0.108 µm. However, some from among the 16 PAHs had significantly higher concentrations at HW than at UB. The concentration of PM-bound BaP at HW was almost twice that for UB (4.3 and 2.3 ng/m^3^, respectively). The significantly higher PM_0.03-1.6_-bound BaP concentration at HW than at UB allows for consideration of BaP as a marker for exhaust emissions in urban areas of Upper Silesia in the periods of lower municipal emissions.

The concentrations of coarser PM (D_p_ > 4.4 µm) and of some its components (OC, EC, Na^+^, K^+^, Cl^−^, Ca^2+^, Al, Si, Fe, Cu, Zn, Mo) also were higher at HW. For example, the average concentrations of PM_4.4-6.8_-, PM_6.8-10_-, and PM_10-40_-related Na^+^ and K^+^ were almost four times greater at HW than at UB, while that for Ca^2+^ was approximately two-fold greater.

The PM mass size distribution was bimodal at UB. At HW, in 6.8-10 µm, the third peak for its density function appeared. Also the maxima of the density functions for Na^+^, K^+^, Cl^−^, and Ca^2+^ occurred in this interval. The coarse ambient particles containing OC, EC, Na^+^, K^+^, Cl^−^, Ca^2+^, Al, Si, Fe, Cu, Zn, Mo are probably a mixture of the particles of worn tires, brake linings, chassis, other car parts, road surface, soil, etc. (Wahlin et al. 2006; Thorpe and Harrison 2008; Pant and Harrison 2013). Such particles, involved in the process of alternate lifting (by passing cars, wind, etc.) and deposition, can stay within the road vicinity for a long time.

The chemical mass closure of PM was checked at both points. There were six groups of identified chemical PM components, and the seventh one, unidentified matter (UM); their masses together made the total PM mass. The mass [EC] of elemental carbon (EC) was assumed to be the analytically determined mass [EC]_A_ of elemental carbon: [EC] = [EC]_A_. The mass [OM] of organic matter (OM, all PM-bound organic compounds) was assumed to be 1.4 of the analytically determined mass [OC]_A_ of organic carbon (OC): [OM] = 1.4[OC]_A_. Secondary inorganic aerosol, SIA, consisted of SO_4_^2−^, NO_3_^−^, NH_4_^+^; [SIA] = [SO_4_^2−^]_A_ + [NO_3_^−^]_A_ + [NH_4_^+^]_A_. [NaCl] = [Cl^−^]_A_ + [Na^+^]_A_.

The rest of the chemical components of PM listed in Table 1, i.e. the analytically determined PM-bound chemical elements (including K^+^, Ca^2+^, and Mg^2+^), were used to define crustal matter (CM) and trace elements (TE). The contents of CM and TE depended on both the PM fraction and the measuring point. These PM-bound elements were divided into two groups based on their enrichment factors EF (Rogula-Kozłowska et al. 2013; 2014). EF expresses how anthropogenic an element is: the closer an element’s EF value is to 1, the smaller the anthropogenic contribution to the element’s concentration in air.

The elements with EF ≤ 20 were assumed crustal (of natural origin). The chemical compounds containing an element with EF ≤ 20 that were known to contribute significantly to the PM mass were included in CM; if there were no such compounds (known) for an element, or the element was probable to occur in numerous compounds, only the element was included in CM. CM included CO_3_^2−^, SiO_2_, Al_2_O_3_, Mg^2+^, Ca^2+^, K_2_O (except PM_0.4-0.65_- and PM_0.06-0.108_-bound K_2_O at UB), FeO and Fe_2_O_3_ (except PM_0.4-1.6_-bound FeO and Fe_2_O_3_ at both points), Rb (except PM_0.17-1.6_-bound Rb at both points), Sr (except PM_0.4-1.6_- and PM_0.03-0.26_-bound Sr at HW, and PM_0.03-1_-bound Sr at UB), PM_2.5-6.8_-bound Ba, and Ba from particles with D_p_ greater than 10 µm. Except for CO_3_^2−^, the masses of the compounds (oxides) in CM (SiO_2_, Al_2_O_3_, K_2_O, FeO and Fe_2_O_3_) were computed stoichiometrically from the masses of their component elements determined analytically; Fe was assumed to be distributed equally between FeO and Fe_2_O_3_. [CO_3_^2−^] was computed from [Ca^2+^]_A_ and [Mg^2+^]_A_ (Marcazzan et al. 2001).

The chemical elements with EF > 20 were assumed to be anthropogenic; these many-source origin elements occurred in many PM-bound compounds that were not determined because of their multiplicity. These elements alone were put in TE. TE consisted of K^+^ (only for PM_0.4-0.65_ and PM_0.06-0.108_ at UB), Sc, Ti, V, Cr, Mn, Fe (Fe at both points for PM_0.4-1.6_ only), Co, Ni, Cu, Zn, As, Se, Br, Rb (Rb at both points for PM_0.17-1.6_ only), Sr (only for PM_0.4-1.6_ and for PM_0.03-0.26_ at HW and for PM_0.03-1_ at UB), Mo, Ag, Cd, Sb, Te, Ba (Ba only for PM_0.03-2.5_ and PM_6.8-10_ at both points), and Pb. The mass [TE] of TE was assumed to be the sum of all the analytically determined masses of the elements from TE.

The mass of unidentified matter [UM] was the deficient mass; it was the difference between the gravimetrically determined mass of PM and [SIA] + [EC] + [OM] + [NaCl] + [CM] + [TE].

**Fig. 3:**
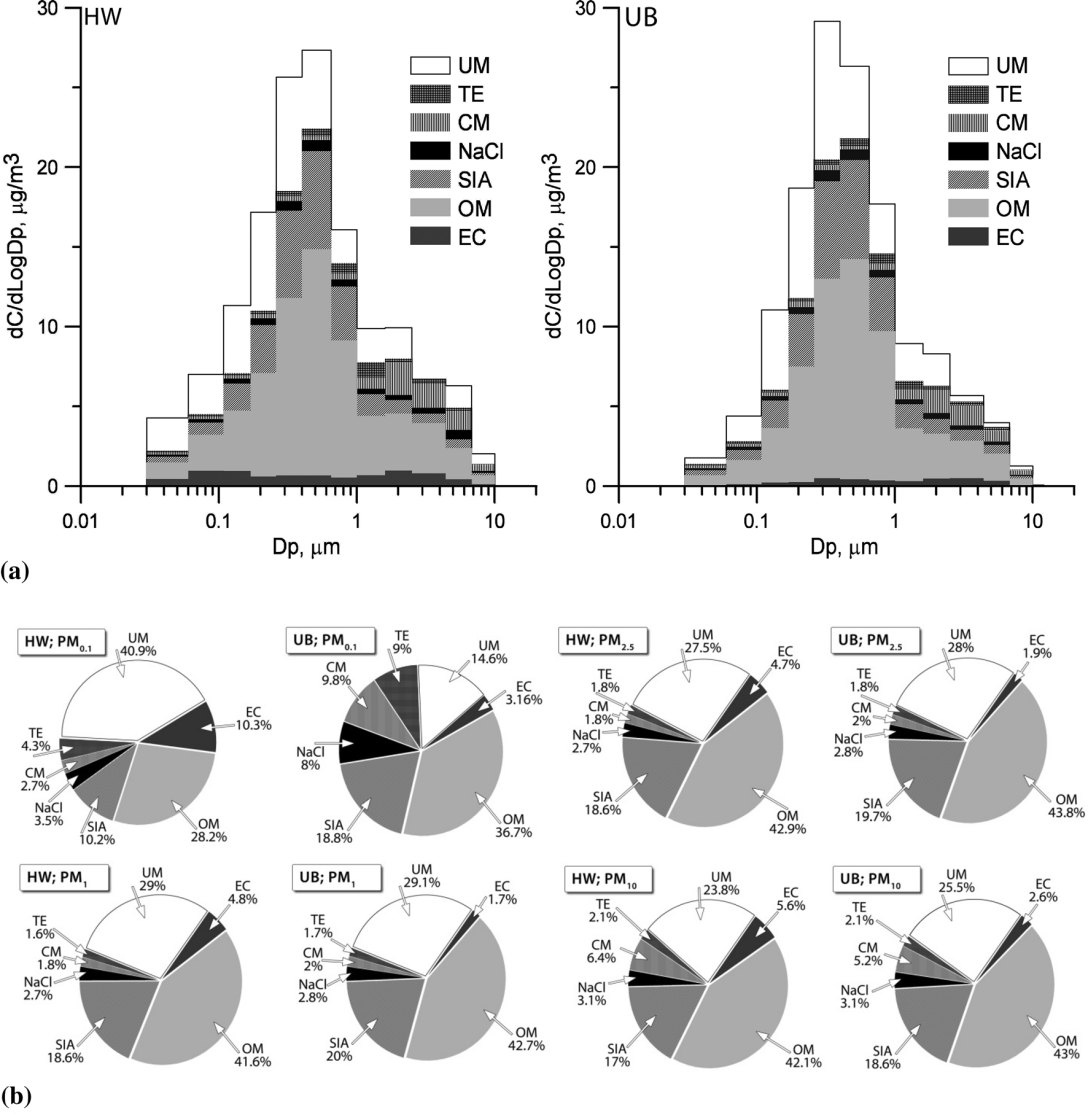
Mass size distribution of the PM component groups (**a**) and the mass reconstruction of selected PM fractions (**b**) at traffic affected HW and at urban background UB (*EC* elemental carbon, *OM* organic matter, *SIA* secondary inorganic aerosol, *NaCl* sum of Na^+^ and Cl^−^, *CM* crustal matter, *TE* trace elements, *UM* unidentified matter)

In Fig. 3, the mass distributions of the PM component groups (relative to particle size) at both sites are presented. Within particular PM fractions, the identified matter accounted for the mass in 52 %–98 % at HW and in 55 %–96 % at UB. For all fractions except PM_4.4-6.8_, the mass of identified compounds was higher at UB than at HW.

The shares of UM in twelve PM fractions were greater, and in ultrafine PM much greater, at HW than at UB (Fig. 3). At HW, the UM shares in PM_0.06-0.65_ and in particles with D_p_ > 10 µm were greatest; whereas at UB, they were greatest in PM_0.108-0.65_ and PM_1.6-4.4_. UM consists of the organic compounds and nitrates that evaporate during transportation and storage of PM samples, unidentified compounds, and water (Tsyro 2005). Also, inaccurate identification of compounds in OM, CM and TE affects UM. For example at HW, considering the high UM content of 12 fractions, [OM] should probably have been computed by multiplying [OC]_A_ by a coefficient greater than 1.4 (Turpin and Lim 2001).

The EC concentrations were higher at HW, and the EC mass distribution differed from that at UB (Table 1; Fig. 2). Therefore, the EC contributions to particular PM fractions, especially to finer ones, at HW were higher than at UB (Fig. 3). The fractional mass contributions of OM to PM at both points were close, and only for the sub-fractions of PM_2.5-6.8_ were they slightly higher (by 5 % in average) at HW.

Although the concentrations of the components of SIA (SO_4_^2−^, NO_3_^−^, NH_4_^+;^ Table 1) at both points were comparable, the distributions of SIA and its mass contributions to the fractions differed slightly between the points. The contributions to PM_0.03-0.108_ and coarser particles (D_p_ > 4.4 µm) were on average greater by 4 and 5 % at UB, respectively.

NaCl was more abundant at HW than at UB only in PM_4.4-10_. This was due to the previously mentioned differences in the ambient concentrations of Na^+^ and Cl^−^ between the points. Similarly, the elevated concentrations of the components of CM, especially of K^+^, Ca^2+^, Al, Si, and Fe, caused a slightly greater share of CM in PM_1.6-10_ at HW (CM contribution to the PM mass at UB was greater than at HW by no more than 2 %). The mass contribution of TE to PM_0.65-1.6_ was greater by about 2 % at HW than at UB. The NaCl, CM, and TE contributions to fine, especially to ultrafine, PM were higher at UB than at HW because ultrafine PM had noticeably lower concentrations at UB (Fig. 3). NaCl and TE contain elements coming from energy production, traffic or industry at UB, and mainly from traffic (both exhaust and non-exhaust emissions) at HW (Pant and Harrison 2013; Kumar et al. 2013; Rogula-Kozłowska et al. 2014). Also CM in ultrafine PM, despite being “crustal,” probably contains particles coming from exhaust gases and furnaces, including particles containing metal oxides from condensation (nuclei mode) (Geller et al. 2006; Maricq 2007).

The differences in the chemical composition of PM between the points were mainly in ultrafine and coarse PM. The distributions of the mass among the component groups differed extremely for PM_0.1_ (in fact PM_0.03-0.108_) between the two points (Fig. 3). PM_1_ and PM_2.5_ (in fact PM_0.03-1_ and PM_0.03-2.5_) differed between the points only in their EC content; PM_10_—in the EC and, greater by about 1 % at HW, CM contents (Fig. 3). The concentration of PM_0.1_ was two times greater at HW than at UB (1.5 and 0.7 µg/m^3^), while the concentrations of PM_2.5_ and PM_10_ at HW were greater by no more than 5 % (for PM_10_ they were 28.6 µg/m^3^ at HW and 27.3 µg/m^3^ at UB).

So, although the number of cars per person grows closer to the European average, traffic emissions are not as significant a PM source in the Silesian Agglomeration as they are in other European regions (e.g. Viana et al. 2008). In Katowice, PM_0.17-1.6_ contributes most to the PM mass, and traffic does not affect this PM fraction much. The concentrations and the chemical composition of this PM fraction within the Silesian Agglomeration are formed by municipal emissions, big power plants, and industry. The presented results show that human health is not more at risk near a big highway than some distance away. At both sampling points the carcinogenic equivalent ratios (CEQ) for particular PM fractions were close, both CEQ for total PM were equal to 4.2 µg/m^3^ (Table 1). As elsewhere in Poland, the concentration, chemical composition, and toxicity of PM in the Silesian Agglomeration depend on the season of the year; i.e., upon meteorological conditions and emissions from heat and power production facilities (Pastuszka et al. 2003, Majewski et al. 2011; Rogula-Kozłowska and Klejnowski 2013; Rogula-Kozłowska et al. 2014).

The present pioneering research shows that some differences in the chemical characteristics of PM_4.4-10_ between two sites in such a specific area as the Silesian Agglomeration can be found only by investigating particular fractions of PM_4.4-10_. However, the experiment should rather be considered as a pilot research and should be repeated on a wider scale at other traffic-affected sites, such as crossroads in a city center, street canyons, etc., to generalize the conclusions.

## Footnotes

1 The group of 16 PAHs included: naphthalene (Na), acenaphthene (Ace), acenaphthylene (Acy), anthracene (An), benzo[a]anthracene (BaA), benzo[a]pyrene (BaP), benzo[b]fluoranthene (BbF), benzo[k]fluoranthene (BkF), benzo[g,h,i]perylene (BghiP), chrysene (Ch), dibenzo[a,h]anthracene (DBA), fluoranthene (Fl), fluorene (F), phenanthrene (Ph), pyrene (Py) and indeno[1,2,3-cd]pyrene (IP).
